# Prevalence of Multimorbidity Among School-Aged Children in the Yangzhou District of China

**DOI:** 10.3390/healthcare13111320

**Published:** 2025-06-02

**Authors:** Jinhan Wang, Qian Zhou, Ying Zhang, Zhuoqi Lai, Weiwei Zhu, Jun Jia, Yongquan Yu, Lihong Yin

**Affiliations:** 1 Key Laboratory of Environmental Medicine Engineering, School of Public Health, Southeast University, Ministry of Education, Nanjing 210009, China; 220223622@seu.edu.cn (J.W.); 230229470@seu.edu.cn (Q.Z.); 101300315@seu.edu.cn (Y.Z.); 220223707@seu.edu.cn (Z.L.); yyqaion@sina.com (Y.Y.); 2 Yangzhou Center for Disease Control and Prevention, Yangzhou 225000, China; zhuweiwei3066@126.com; 3 Jianye District Center for Disease Control and Prevention, Nanjing 210019, China; junjcdc@163.com

**Keywords:** multimorbidity, school-aged children, cross-sectional study

## Abstract

**Background:** Health issues among school-age children have emerged as a global public health concern. These conditions often do not occur in isolation but tend to cluster, indicating a widespread issue of multimorbidity among this population. This study examined the prevalence and clustering of multimorbidity among school-aged school students in the Yangzhou district. **Methods**: A repeated cross-sectional analysis was conducted from 2019 to 2024, including 22,512 students aged 6–18 years. Common diseases, under national key monitoring, including myopia, dental caries, obesity, elevated blood pressure, and growth disorders, were assessed. Multimorbidity patterns were identified using association rule mining (Apriori algorithm) with predefined thresholds (support ≥ 2.0%, confidence ≥ 20.0% and lift > 1). **Results**: The multimorbidity prevalence among school-age students in the Yangzhou district is 53.95%. The most frequent multimorbidity was found in dental caries and myopia, while the most common ternary pattern was found in obesity, dental caries, and myopia. The following gender differences were observed: boys had a higher multimorbidity prevalence (56.4%) compared to girls (51.2%), with boys more likely to exhibit obesity and dental caries, while girls showed a higher prevalence of myopia-related multimorbidity. By educational stage, primary school students showed a multimorbidity rate of 50.3%, junior high showed a rate of 54.6%, and senior high showed a rate of 57.9%, indicating a rising trend across age groups. Patterns of multimorbidity varied but were interrelated. **Conclusions**: From 2019 to 2024, the prevalence of multimorbidity among school-aged children in Yangzhou remained relatively high, primarily manifesting as co-occurring myopia and other health issues. Patterns of multimorbidity across gender and educational stage varied but were interrelated.

## 1. Introduction

School-age children’s health has emerged as a global public health priority since the 21st century. The physical and mental well-being of primary and secondary students—a population that undergoes crucial developmental stages—not only influences individual growth trajectories but also constitutes the foundation of national human capital development [1]. Over the past two decades, school-aged children have faced a convergence of health risks, including myopia, obesity, and psychological disorders [2]. Globally, these conditions contribute significantly to disability-adjusted life years (DALYs) and reflect shifting pediatric health burdens in both developed and developing contexts [3]. While developed countries report rising rates of adolescent obesity and mental health issues [4], developing nations often contend with the dual burden of undernutrition and emerging lifestyle-related disorders [5,6]. In East Asia, similar pediatric health challenges are observed. For instance, Japan reports a myopia prevalence of approximately 50% among junior high school students, while South Korea has documented adolescent obesity rates exceeding 18% [7,8]. China’s status as the nation with the largest school-age population (193 million students in 2023) coincides with concerning health indicators surpassing international benchmarks, including myopia prevalence (52.7%) and scoliosis detection rates (2.8%) [9,10,11]. These pediatric health conditions demonstrate the following multidimensional impacts: direct developmental consequences (e.g., 30-fold elevated blindness risk from severe myopia [12]), substantial healthcare cost escalations (14.2% annual per capita expenditure increase among Chinese students [13]), and productivity losses through school absenteeism (8.3% illness-related absence rate [14]). Longitudinal studies further indicate that childhood health impairments may predispose individuals to adult-onset chronic diseases, metabolic dysregulation [15,16], immune deficiencies, and cognitive sensory deficits [17,18,19]. This evidence underscores the imperative need for enhanced childhood health interventions to disrupt the trajectory from pediatric morbidity to adult health deterioration.

The evolving complexity of disease ecology has transformed clinical presentations from singular disorders to multimorbidity clusters, necessitating fundamental reforms in traditional single-disease prevention paradigms. School-aged populations now demonstrate an alarming multimorbidity prevalence exceeding 20% globally. In the United States, national health surveys identify the following distinct multimorbidity patterns: 11.5% of children exhibit the obesity–asthma–sleep disorder triad, while obese children show a 3.2-fold greater prevalence of elevated blood pressure (19.5% vs. 6.1% in normal-weight peers) according to CDC/NCHS data [20,21]. European cohort studies document that psycho-somatic multimorbidity reduces academic performance by 47% [22], and multinational meta-analyses confirm obese children’s 67% higher asthma risk compared to normal-weight children (relative risk = 1.67; 95% CI: 1.52–1.83) [23]. Chinese epidemiological studies reveal syndemic interactions between nutritional deficiencies and oral health, with malnourished children showing a 2.1-fold increased dental caries risk [24]. This multi-system health crisis is intensifying temporally. Pediatric obesity rates have accelerated 38% faster than adult rates since 2010 [25], predisposing youth to early-onset metabolic syndrome, type 2 diabetes, and cardiovascular comorbidities [26]. Longitudinal evidence confirms that childhood obesity increases adult hypertension risk by 41% (95% CI: 1.32–1.51) [27] and cardiovascular mortality by 29% [28]. These findings collectively highlight the critical imperative for multidimensional intervention strategies to combat the escalating pediatric multimorbidity crisis. In this study, multimorbidity is defined as the co-occurrence of two or more chronic health conditions within an individual, consistent with widely accepted epidemiological definitions.

Despite these trends, the existing literature remains predominantly adult-focused [29], with limited attention paid to multimorbidity patterns among younger populations. This underscores the need for targeted epidemiological investigations in children, particularly within rapidly urbanizing regions such as Eastern China. By building upon this epidemiological context, we conducted a cross-sectional study investigating the multimorbidity patterns among primary and secondary school students in Yangzhou district from 2019 to 2024. This research aimed to quantify the prevalence of multimorbidity, characterize dominant disease clusters, and identify potential determinants through multivariate analysis, thereby providing evidence-based recommendations for preventive strategy development.

## 2. Materials and Methods

### 2.1. Study Design and Participants

Yangzhou was selected due to its well-established school health surveillance infrastructure and comprehensive local CDC collaboration, enabling consistent, high-quality data collection across years. As a mid-sized city in Eastern China with above-average socioeconomic indicators, it represents urbanizing regions with increasing health burdens but adequate health system capacity.

A population-based repeated cross-sectional study was conducted among school-aged children (6–18 years) in Yangzhou to investigate temporal trends in pediatric multimorbidity from 2019 to 2024. Stratification was conducted based on school level (primary, junior high, senior high) and geographic subdistricts within Yangzhou (urban, peri-urban, and rural zones). Within each stratum, schools were randomly selected in proportion to the total student population in that area to ensure demographic representativeness. At the school level, all eligible students that met the inclusion criteria were invited to participate. This approach aimed to capture socioeconomic and environmental diversity across the Yangzhou district. The annual cohorts comprised 3887 (2019), 4026 (2020), 4099 (2021), 3743 (2022), 3894 (2023), and 3988 (2024) students, respectively (total N = 23,637; mean annual participation = 3.94 ± 140).

Enrollment criteria required (1) continuous residency in the study area for ≥2 years, and (2) the availability of complete health screening records. Exclusion criteria eliminated participants with missing demographic variables (3.2%) or incomplete diagnostic data (1.7%), yielding an analytic sample of 22,512 students (retention rate = 95.2%). Beyond the initial exclusion of incomplete records, data underwent preprocessing procedures, including outlier detection using boxplot analysis, logical consistency checks (e.g., BMI within biologically plausible ranges), and duplicate entry removal. All datasets were cross-validated annually through school-level quality audits coordinated by the local CDC.

### 2.2. Variable Definition

This study included the following common diseases of students under national key monitoring: obesity, wasting, stunting, myopia, dental caries, hepatitis, nephritis, heart disease, elevated blood pressure, anemia, diabetes mellitus, and allergic asthma. The diagnostic criteria for all included conditions were based on standardized national screening protocols issued by the Ministry of Education and National Health Commission of China. Detailed diagnostic thresholds, including age- and sex-specific BMI cutoffs for obesity and standard visual acuity thresholds for myopia, are presented in Supplementary Table S1.

Non-diagnosed conditions were coded as ‘0’ and diagnosed conditions as ‘1’. The summation of all conditions yielded a total morbidity score. Participants with ≥2 concurrent common diseases were classified as having multimorbidity, while those with 0–1 were categorized as non-multimorbidity. Additional variables incorporated in this study included sociodemographic factors (gender, grade).

Diagnostic consistency was ensured through the annual calibration of measurement devices, the centralized training of school physicians using WHO STEPS protocols, and blinded dual-entry validation for 10% random samples. All diagnoses were conducted by certified school physicians as part of the annual physical examination mandated by the local education and health authorities. Diagnoses were based on standardized national health screening protocols issued by the Chinese Ministry of Education and Ministry of Health. No self-reported or parent-reported data were used. Mental health conditions, including anxiety and depression, were not included in the analysis due to the absence of standardized psychological screening protocols across all participating schools. Additionally, self-reported or parent-reported data for such conditions were not collected consistently, precluding their inclusion.

### 2.3. Statistical Analysis

The research methods for multimorbidity include disease counting [30], cluster analysis [31], factor analysis [32], and association rule analysis. This study conducts the association rule analysis of common diseases using the Apriori algorithm in R version 4.2.1, which has been applied in related fields. Through the Apriori algorithm, we demonstrate the associations between diseases in multimorbidity patterns and quantify the strength of these associations using numerical values. Three kernel values are involved with association rule analysis, such as support, confidence, and lift. Based on this study, the support of A→B was the probability of the simultaneous occurrence of common diseases A and B. The confidence was the conditional probability of suffering from common diseases B under the premise of suffering from common diseases A. The degree of lift reflects the influence of the consequent B on the antecedent A compared to the overall. The analysis follows the following predefined thresholds: a minimum support level of 2.0%, a minimum confidence threshold of 20.0%, and lift is greater than 1. SPSS 26.0 was used for sorting and screening data. Descriptive statistics were performed by using R version 4.2.1.

For instance, if 30% of students have both myopia and dental caries, this gives a support of 30%. If 60% of students with dental caries also have myopia, then the confidence of the rule “Dental Caries→Myopia” is 60%. If this co-occurrence is 1.2 times more likely than expected under independence, the lift is 1.2.

## 3. Results

### 3.1. Description of the Participants

The survey encompassed a total of 22,512 students from primary and secondary school. The annual participation figures were as follows: 3747 students in 2019, 3782 in 2020, 3830 in 2021, 3637 in 2022, 3690 in 2023, and 3826 in 2024. Comprehensive demographic data are delineated in Table 1.

### 3.2. Prevalence of Diseases

**Figure 1:**
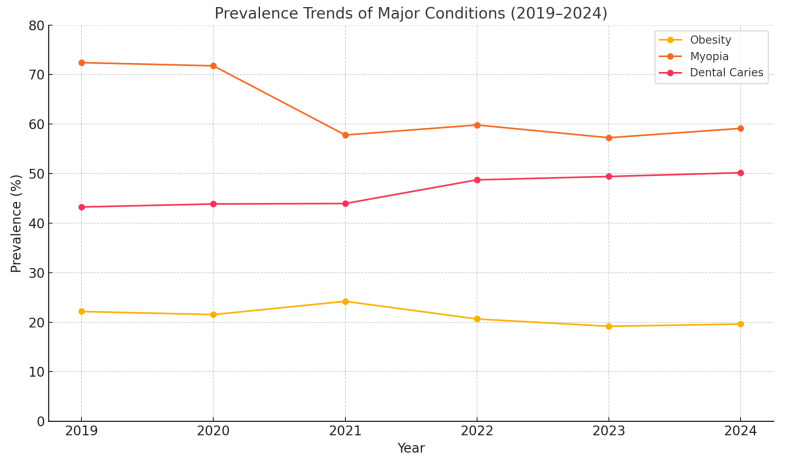
Prevalence of obesity, myopia, and dental caries among school-aged children in the Yangzhou District from 2019 to 2024.

Table 2 presents disease prevalence (%) from 2019 to 2024. Between 2019 and 2024, the most notable trend was a decline in myopia prevalence from 72.41% to 57.25%, likely reflecting intensified public health interventions. Dental caries prevalence steadily increased, exceeding 50% by 2024, while obesity showed modest fluctuations, peaking in 2021. Less common conditions, such as hepatitis, nephritis, and heart disease, only emerged in later years, albeit at low prevalence levels (<0.15%), potentially indicating improved diagnostic capture or environmental exposures. Notably, the prevalence of allergic asthma showed a sharp increase in 2023 (1.21%) compared to prior years (<0.12%). This rise may be due to improved diagnostic reporting, expanded asthma screening efforts, or environmental factors such as post-COVID changes in air quality or school attendance patterns. Further investigation is required to confirm the cause of this spike (Figure 1). There were trends in the prevalence of obesity, myopia, and dental caries among school-aged children in the Yangzhou District from 2019 to 2024. While myopia showed a general decline, dental caries steadily increased. Obesity fluctuated with a peak in 2021.

To assess the potential impact of excluding 4.9% of participants with incomplete data, a sensitivity analysis was conducted. We re-ran the primary multimorbidity prevalence analysis by including participants with partial diagnostic data using available-case analysis. For multimorbidity classification, individuals were considered multimorbid if ≥2 conditions were confirmed among available diagnoses. No significant differences were observed in overall prevalence or dominant disease clusters.

### 3.3. Multimorbidity Patterns

The analysis selected common disease survey results of school-aged students from 2019 to 2024 in the Yangzhou district. The prevalence rate of multimorbidity among students was 53.95%, with 35.49% having two concurrent conditions, 10.03% having three conditions, 1.85% having four conditions, and 0.10% presenting five or more conditions. The prevalence patterns of student multimorbidity are detailed in Table 3.

The temporal analysis of major multimorbidity patterns revealed notable shifts across the study period. The myopia and dental caries cluster, while consistently dominant, increased from 29.2% in 2019 to 34.7% in 2024, suggesting an intensifying co-occurrence of these conditions. In contrast, the obesity and dental caries combination showed a modest decline after peaking in 2021. These trends may reflect changing lifestyle and behavioral patterns among school-aged children and underscore the need for time-sensitive, integrated health strategies.

A sensitivity analysis incorporating the 4.9% of students with partial data yielded a multimorbidity prevalence of 53.68%, compared to 53.95% in the complete-case analysis. The top multimorbidity patterns remained unchanged (e.g., myopia and dental caries). This supports the robustness of the primary findings despite minimal data exclusion.

### 3.4. Multimorbidity Analysis

Using predefined thresholds of minimum support 2.0% and minimum confidence 20.0%, we identified the top 10 association rules with the highest support values, where myopia-related multimorbidity patterns demonstrated the highest frequency. Higher support values indicated more prevalent disease patterns, with the three most frequent multimorbidity clusters being dental caries and myopia, obesity and myopia, and elevated blood pressure and myopia. The three strongest associations were identical to these prevalent patterns. For example, 32.45% of students exhibited coexisting dental caries and myopia, which is a strikingly high prevalence that suggests a strong comorbidity between vision and oral health. This finding underscores the need for integrated preventive measures targeting both conditions, especially during school-age development. Detailed results are presented in Table 4.

### 3.5. Gender-Stratified Multimorbidity

Gender-stratified association rule mining was conducted using identical thresholds (minimum support 2.0%, minimum confidence 20.0%), selecting the top 10 rules by support. Across both genders, the most prevalent multimorbidity pattern was dental caries and myopia. Among males, the three most frequent chronic disease clusters were dental caries and myopia, obesity and myopia, and dental caries and obesity. The strongest associations in males emerged as elevated BP and myopia, dental caries and myopia, and obesity and myopia. For females, the predominant patterns were dental caries and myopia, obesity and myopia, and elevated BP and myopia, with the strongest associations being dental caries and myopia, elevated BP and myopia, and the triad dental caries, obesity, and myopia. Detailed gender-specific results are compiled in Table 5. The overall multimorbidity prevalence was significantly higher in males (56.4%) than females (51.2%, *p* < 0.001). The most common pattern among males was obesity and dental caries, while females showed a higher proportion of myopia and dental caries. Gender-based differences in the frequency of these patterns were statistically significant (*p* < 0.01), as shown in Supplementary Table S2.

### 3.6. Educational-Stage Multimorbidity

Educational stage-stratified association rule mining was performed using consistent thresholds (minimum support 2.0%, minimum confidence 20.0%), prioritizing rules by support magnitude. For primary school students, the three most prevalent multimorbidity patterns were myopia and dental caries, dental caries and obesity, and myopia and obesity. The strongest associations manifested as dental caries and myopia, obesity, dental caries, and myopia, and obesity and dental caries. Among middle school students, dominant patterns included dental caries and myopia, obesity and dental caries, and obesity and myopia, with the strongest rules being dental caries and myopia, obesity and myopia, and elevated BP and myopia. High school cohorts exhibited the patterns dental caries and myopia, myopia and obesity, elevated BP and myopia, with peak associations at elevated BP and myopia, obesity and myopia, and dental caries and myopia. Comprehensive results are cataloged in Table 6.

## 4. Discussion

Notable temporal trends were observed across the five-year study period. Myopia prevalence declined consistently from 72.41% in 2019 to 57.25% in 2023 before plateauing in 2024, potentially reflecting national myopia control policies. Conversely, dental caries prevalence showed a steady upward trajectory, rising from 43.26% to 50.18%, suggesting insufficient improvement in oral hygiene behaviors during the same period. Obesity fluctuated, peaking in 2021 (24.21%) and declining thereafter, indicating the potential short-term effects of health interventions or lifestyle shifts.

People with multimorbidity often face a greater risk of poor health, and disease prevention and management are more complex and urgent [33]. Adequate multimorbidity information and good multimorbidity management can improve the quality of life of people with multimorbidity and reduce medical costs [34]. This study found that a high proportion of school-aged students in Yangzhou district were suffering from multiple common diseases simultaneously. Myopia had the most comorbidity patterns, accounting for six types. A study conducted on school-aged students in Guangdong Province revealed that obesity and myopia are the most prevalent multimorbidity patterns among this demographic [35]. A survey from 2019 to 2022 focusing on students in Inner Mongolia identified the top three multimorbidity patterns as obesity and myopia, elevated blood pressure and myopia, and depression and myopia [36]. These findings are similar to the results of our study, highlighting myopia’s significant prevalence and severity as a common disease among school-aged students. Additionally, a survey on students in Tianjin indicated that the most frequent multimorbidity patterns are obesity and myopia and obesity and elevated blood pressure [37], and it also underscored the severity of the obesity issue. The relatively high multimorbidity prevalence reported in this study may partly result from broad inclusion criteria, particularly conditions like mild myopia or early-stage dental caries, which are prevalent but may not always lead to functional impairment. While these conditions were diagnosed based on standardized criteria, their inclusion raises questions about possible overdiagnosis, especially in the context of school-based mass screening programs. Nevertheless, their public health relevance should not be understated, as even mild presentations can affect learning and quality of life and may progress without intervention. Future studies may consider applying severity thresholds or functional impairment indices to better classify clinically significant multimorbidity.

This survey found that the most common multimorbidity patterns among both boys and girls were dental caries and myopia. The multimorbidity patterns of obesity and dental caries were more strongly correlated among boys, probably because boys usually consume more high-calorie, high-fat foods than girls and pay less attention to weight management; thus, the obesity rate is usually higher [38]. Girls tend to pay more attention to dental cleaning during childhood, while boys are relatively lax in oral health management. The multimorbidity pattern of myopia was also the most common among girls, which was probably closely related to outdoor activity time. Previous studies have shown that boys usually spend more time outdoors than girls, which may reduce the risk of myopia in boys to a certain extent [39]. The clustering of conditions such as myopia, obesity, and dental caries likely reflects a set of shared behavioral and environmental risk factors, including high intake of sugary foods and beverages, prolonged screen exposure, low physical activity levels, and insufficient outdoor time. Studies have shown that sugar-sweetened beverage consumption is associated with both increased caries risk and adiposity in children [40], while sedentary behaviors contribute to both visual strain and metabolic dysfunction [41]. These overlapping exposures may drive the observed multimorbidity patterns.

Multimorbidity patterns vary in different stages of education, but they are interrelated. In this study, dental caries were found in the three most common multimorbidity patterns and the three most strongly correlated multimorbidity patterns among primary school students. This shows that the oral hygiene of young students should be paid more attention to avoid affecting their oral health in adulthood. Because children are still in the developmental stage, their immune systems are not fully mature, and their teeth have poor self-cleaning ability, resulting in a high prevalence of dental caries in deciduous teeth in children [42]. Studies have shown that dental caries in children’s deciduous teeth will change their eating habits and affect their growth and development [43]. The health of children’s deciduous teeth directly affects the health of permanent teeth in adulthood. This may be one of the reasons why dental caries and myopia are strongly correlated in junior high school students in this study. In addition, this study found that the proportion of junior high school students suffering from both dental caries and myopia is relatively high. In addition to bad eye habits, unhealthy dietary intake also affects the occurrence of myopia. Students who often drink sugary drinks will affect their eye development, leading to myopia [44]. Sugary drinks are also one of the causes of common diseases among school-aged students, such as obesity and dental caries. Obesity has been proven to be a risk factor for myopia [45]. This pattern may reflect shared risk factors between myopia and dental caries—such as dietary habits or lifestyle behaviors—though causal relationships cannot be inferred due to the study’s cross-sectional design. The increased prevalence and correlation of the multimorbidity pattern of myopia and elevated blood pressure in high school students may be related to the increased academic pressure in high school, the reduced time for extracurricular activities, and the increased psychological pressure of students [46].

We conducted a temporal stratification of the top five multimorbidity patterns to assess whether their composition and prevalence shifted over time. Notably, as myopia prevalence declined from 72.41% in 2019 to 57.25% in 2023, the frequency of myopia and obesity and myopia and elevated BP combinations also decreased by approximately 4–5 percentage points. Conversely, dental caries and obesity remained relatively stable across years, suggesting more persistent behavioral and dietary risk factors. These observations indicate that shifts in single disease prevalence (e.g., myopia) can reshape the broader multimorbidity landscape.

Although a small proportion (4.9%) of participants were excluded due to incomplete data, sensitivity analysis indicated negligible impact on prevalence estimates or disease pattern identification. This study has certain limitations. While mental health conditions such as depression and anxiety are increasingly recognized in pediatric multimorbidity research, they were not included in the present study due to the lack of standardized, school-based mental health screening in the surveillance system during the study period. Future research should prioritize integrating validated mental health assessment tools to better capture psychosocial dimensions of multimorbidity in children and adolescents. Additionally, this study focuses solely on the Yangzhou district in Eastern China, and the results cannot be generalized to the common multimorbidity among school-aged students nationwide. Future studies should incorporate the common multimorbidity of school-aged students from various regions across the country; this study is a cross-sectional survey that only reflects data at a specific moment and cannot dynamically monitor the common multimorbidity among school-aged students. Future studies should establish a cohort study on the common multimorbidity of school-aged students in China to dynamically monitor the multimorbidity. Further research is warranted to explore the underlying determinants of these multimorbidity trends and to assess the effectiveness of early intervention strategies in reducing long-term health risks. It is also important to acknowledge that several potential confounding factors were not included in the current analysis. Variables such as socioeconomic status, dietary intake, physical activity, screen exposure time, and parental education could substantially influence the development and clustering of health conditions among school-aged children. Future studies should incorporate these variables to better isolate the independent effects of disease combinations and clarify underlying mechanisms.

## 5. Conclusions

This study provides new evidence on the high burden and demographic patterns of multimorbidity among school-aged children in Eastern China. The observed clustering of vision and oral health problems, particularly the comorbidity of myopia and dental caries, highlights the need for integrated screening programs within school settings. Policymakers should consider implementing joint dental-vision checkups as part of routine physicals, especially in urban districts with high academic pressure. Moreover, the gender-specific patterns suggest that health education interventions should be tailored, such as visual hygiene campaigns for girls and oral-nutrition education for boys. Future longitudinal research should explore causal mechanisms and assess the long-term impact of early interventions on multimorbidity trajectories.

## Figures and Tables

**Table 1:** Demographic characteristics of the population from 2019 to 2024.

Demographic Characteristics		2019	2020	2021	2022	2023	2024
Gender	Male	1992	1907	1786	1833	1774	1977
	Female	1755	1875	1903	1804	1928	1866
Educational Stage	Primary School	1830	1792	1829	1845	1829	1879
	Junior High School	964	913	931	903	938	977
	Senior High School	953	1077	905	889	923	970
Total		3747	3782	3830	3637	3690	3826

**Table 2:** Prevalence of common diseases with 95% confidence intervals (2019–2024).

Diseases	2019	2020	2021	2022	2023	2024	Total
Obesity	22.15% (20.82–23.48%)	21.53% (20.22–22.84%)	24.21% (22.85–25.57%)	20.64% (19.32–21.96%)	19.17% (17.90–20.44%)	19.62% (18.36–20.88%)	21.23% (20.70–21.77%)
Wasting	3.81% (3.20–4.42%)	3.73% (3.13–4.33%)	2.32% (1.84–2.80%)	3.31% (2.73–3.89%)	2.56% (2.05–3.07%)	3.07% (2.52–3.62%)	3.13% (2.90–3.36%)
Stunting	0.24% (0.08–0.40%)	0.15% (0.03–0.27%)	0.34% (0.16–0.52%)	0.19% (0.05–0.33%)	0.17% (0.04–0.30%)	0.21% (0.06–0.36%)	0.22% (0.16–0.28%)
Myopia	72.41% (70.98–73.84%)	71.77% (70.34–73.20%)	57.80% (56.24–59.36%)	59.82% (58.23–61.41%)	57.25% (55.65–58.85%)	59.13% (57.57–60.69%)	63.04% (62.41–63.67%)
Dental caries	43.26% (41.67–44.85%)	43.87% (42.29–45.45%)	43.96% (42.39–45.53%)	48.74% (47.12–50.36%)	49.42% (47.81–51.03%)	50.18% (48.60–51.76%)	46.55% (45.90–47.20%)
Hepatitis	0.00% (0.00–0.00%)	0.00% (0.00–0.00%)	0.00% (0.00–0.00%)	0.01% (0.00–0.04%)	0.13% (0.01–0.25%)	0.01% (0.00–0.04%)	0.02% (0.01–0.04%)
Nephritis	0.00% (0.00–0.00%)	0.00% (0.00–0.00%)	0.00% (0.00–0.00%)	0.00% (0.00–0.00%)	0.01% (0.00–0.04%)	0.01% (0.00–0.04%)	0.01% (0.00–0.02%)
Heart disease	0.00% (0.00–0.00%)	0.00% (0.00–0.00%)	0.00% (0.00–0.00%)	0.01% (0.00–0.04%)	0.03% (0.00–0.09%)	0.02% (0.00–0.06%)	0.01% (0.00–0.02%)
Elevated BP	13.92% (12.81–15.03%)	14.21% (13.10–15.32%)	14.44% (13.33–15.55%)	12.68% (11.60–13.76%)	12.25% (11.19–13.31%)	12.57% (11.52–13.62%)	13.35% (12.91–13.80%)
Anemia	0.36% (0.17–0.55%)	0.27% (0.10–0.44%)	0.24% (0.09–0.39%)	0.24% (0.08–0.40%)	0.82% (0.53–1.11%)	0.40% (0.20–0.60%)	0.39% (0.31–0.47%)
Diabetes	0.00% (0.00–0.00%)	0.00% (0.00–0.00%)	0.00% (0.00–0.00%)	0.01% (0.00–0.04%)	0.01% (0.00–0.04%)	0.00% (0.00–0.00%)	0.01% (0.00–0.01%)
Allergic asthma	0.11% (0.00–0.22%)	0.12% (0.01–0.23%)	0.01% (0.00–0.04%)	0.01% (0.00–0.04%)	1.21% (0.86–1.56%)	0.93% (0.63–1.23%)	0.40% (0.32–0.48%)

**Table 3:** Prevalence of multimorbidity among students.

Number of Multimorbidity	2	3	4	≥5	Total
Prevalence (%)	35.49	10.03	1.85	0.10	53.95

**Table 4:** Multimorbidity analysis for common diseases among students.

Antecedent	Consequent	Support (%)	Confidence (%)	Lift
Dental caries	Myopia	32.45	67.89	1.13
Myopia	Dental caries	32.45	45.00	1.13
Myopia	Obesity	14.59	20.24	1.21
Obesity	Myopia	14.59	69.76	1.21
Elevated BP	Myopia	10.97	73.18	1.08
Obesity	Dental caries	9.23	44.13	1.15
Elevated BP	Dental caries	6.81	45.42	1.12
Dental caries, Obesity	Myopia	6.01	65.10	1.05
Myopia, Obesity	Dental caries	6.01	41.19	1.04
Obesity	Dental caries, Myopia	6.01	28.73	1.06

**Table 5:** Multimorbidity analysis for common diseases among students of different genders.

Gender	Antecedent	Consequent	Support (%)	Confidence (%)	Lift
Female	Dental caries	Myopia	36.39	70.69	1.12
	Myopia	Dental caries	36.39	48.79	1.12
	Obesity	Myopia	11.98	71.81	1.31
	Elevated BP	Myopia	11.01	77.40	1.14
	Obesity	Dental caries	8.14	48.81	2.32
	Elevated BP	Dental caries	7.04	49.45	1.98
	Dental caries, Obesity	Myopia	5.39	66.24	1.03
	Myopia, Obesity	Dental caries	5.39	45.02	1.17
	Obesity	Dental caries, Myopia	5.39	32.33	1.34
	Elevated BP, Dental caries	Myopia	5.26	74.69	1.31
Male	Dental caries	Myopia	28.79	64.87	1.12
	Myopia	Dental caries	28.79	41.24	1.12
	Myopia	Obesity	17.02	24.38	1.15
	Obesity	Myopia	17.02	68.48	1.15
	Elevated BP	Myopia	10.94	69.63	1.04
	Dental caries	Obesity	10.25	23.08	1.37
	Obesity	Dental caries	10.25	41.21	1.21
	Elevated BP	Obesity	6.99	44.48	2.31
	Obesity	Elevated BP	6.99	28.10	2.31
	Elevated BP	Dental caries	6.60	42.02	1.14

**Table 6:** Multimorbidity analysis for common illnesses among students of different educational stages.

Educational Stage	Antecedent	Consequent	Support (%)	Confidence (%)	Lift
Primary School	Dental caries	Myopia	32.23	49.43	1.15
	Myopia	Dental caries	32.23	66.06	1.15
	Dental caries	Obesity	14.78	22.19	1.26
	Obesity	Dental caries	14.78	61.39	1.26
	Myopia	Obesity	11.19	22.94	1.23
	Obesity	Myopia	11.19	47.47	1.23
	Obesity	Dental caries, Myopia	7.01	29.75	1.19
	Dental caries, Myopia	Obesity	7.01	21.76	1.19
	Myopia, Obesity	Dental caries	7.01	62.67	1.12
	Dental caries, Obesity	Myopia	7.01	48.45	1.17
Junior High School	Dental caries	Myopia	30.08	68.43	1.11
	Myopia	Dental caries	30.08	44.51	1.14
	Myopia	Obesity	17.37	25.71	1.21
	Obesity	Myopia	17.37	64.06	1.21
	Dental caries	Obesity	10.17	23.13	1.24
	Obesity	Dental caries	10.17	37.50	1.24
	Elevated BP	Myopia	8.89	65.12	1.04
	Obesity	Elevated BP	6.25	23.05	2.16
	Elevated BP	Obesity	6.25	45.74	2.16
	Obesity, Myopia	Dental caries	5.93	34.15	1.05
Senior High School	Dental caries	Myopia	29.91	87.25	1.13
	Myopia	Dental caries	29.91	33.11	1.13
	Obesity	Myopia	13.02	89.41	1.21
	Elevated BP	Myopia	11.48	91.16	1.01
	Obesity	Dental caries	5.14	35.29	1.21
	Obesity	Elevated BP	4.97	34.12	2.31
	Elevated BP	Obesity	4.97	39.46	2.31
	Obesity, Myopia	Elevated BP	4.46	34.21	1.35
	Obesity	Elevated BP, Myopia	4.46	30.59	1.34
	Elevated BP	Obesity, Myopia	4.46	35.37	1.28

## Data Availability

The data presented in this study are available on request from the corresponding author. The data are not publicly available due to privacy.

## Supplementary Materials

The following supporting information can be downloaded at: https://www.mdpi.com/article/10.3390/healthcare13111320/s1. Table S1. Diagnostic Criteria for Common Conditions Included in the Study; Table S2. Gender Differences in Multimorbidity Patterns.

## Abbreviations

CDC	Centers for Disease Control and Prevention
NHANES	National Health and Nutrition Examination Survey
CHILD	Childhood Health, Air Pollution and Lung Development
Elevated BP	Elevated Blood Pressure
