# Supplementary material for: Prevalence of Multimorbidity Among School-Aged Children in the Yangzhou District of China
Source: Healthcare (Basel). 2025 Jun 2;13(11):1320. doi: 10.3390/healthcare13111320 (PMC12154384; doi:10.3390/healthcare13111320)
Supplement: Supplementary file 1 [file healthcare-13-01320-s001.zip › healthcare-3643697-supplementary.pdf]

Supplementary Table S1. Diagnostic Criteria for Common Conditions Included in the Study

Condition	Diagnostic Criteria
Obesity	BMI ≥ age- and sex-specific cutoff based on Chinese Screening Standards (WS/T 586-2018)
Wasting	BMI ≤ 5th percentile for age and sex
Stunting	Height-for-age ≤ 2 standard deviations below WHO growth reference
Myopia	Uncorrected visual acuity < 5.0 (LogMAR ≥ 0.1) in either eye and spherical equivalent ≤ −0.50 D
Dental Caries	Clinically visible decayed, missing, or filled teeth, diagnosed by licensed school dentist
Elevated Blood Pressure	Blood pressure ≥ 95th percentile for age, sex, and height (based on Chinese pediatric BP charts)
Anemia	Hemoglobin concentration < 110 g/L (age 6–11), < 115 g/L (12–14), < 120 g/L (15–18), per WHO guidelines
Allergic Asthma	Self-reported or parent-confirmed physician diagnosis

Supplementary Table S2. Gender Differences in Multimorbidity Patterns

Pattern	Male (n/%)	Female (n/%)	p-value
Myopia & Dental Caries	2800 (41.2%)	3000 (51.7%)	< 0.0001
Obesity & Dental Caries	1900 (27.9%)	1200 (20.7%)	< 0.0001
Myopia & Obesity	1200 (17.6%)	1100 (19.0%)	0.0592
Myopia & Elevated BP	900 (13.2%)	500 (8.6%)	< 0.0001
